# Case Report: Post-operative mitral valve replacement complicating with a large cardiac mass and role of TEE in decision making

**DOI:** 10.12688/f1000research.145007.1

**Published:** 2024-04-22

**Authors:** Narasimha Pai D, Chaithra Nayak, Padmanabh Kamath, Syed Waleem Pasha, Deepa Noronha

**Affiliations:** 1 Department of Cardiology, Kasturba Medical College, Mangalore, Manipal Academy of Higher Education, Manipal, India

**Keywords:** Prosthetic mitral valve, Echocardiography, Reexploration, Cardiac mass, Complication

## Abstract

**Background:**

Postoperative complications are an integral part of valve surgery. Common complications include hematomas, bleeding, valve dehiscence, paravalvular leak, and acute PV thrombosis. With the available data from published articles, the rate of all valve-related complications is 0.7 to 3.5% per patient annually. [1] The pathology involved is multifactorial, often blood vessel injury leading to bleeding and hematoma. Although postoperative complications are evident, incidental diagnosis of a cardiac mass in an asymptomatic and hemodynamically stable patient postoperatively is crucial, requiring non-invasive imaging for immediate surgical action.

**Case presentation:**

A woman in her 50s presented with chief complaints of worsening dyspnoea with suddenonset and chest pain. Clinical findings showed apex shifted downward and outward, wide split S2, and a mid systolic murmur radiating to the mid axillary line. Twelve-lead ECG showed LA enlargement, that aligned with X-ray findings. 2D Echocardiography revealed MVP with severe MR and a dilated LV. The patient underwent successful mitral valve replacement as per ACC/AHA class I recommendation. However, postoperative TTE showed a remarkably large mass measuring 5.6 cm*4.6 cm in the RA. Reexploration was performed, followed by mass excision. Plenty of organized clots were seen compressing the RA. TEE showed no evidence of mass. Following stabilization,the patient was discharged considering optimal INR values and prosthetic valve function assessed by echocardiography. The patient’s symptoms improved during the first follow-up.

**Conclusion:**

Although postoperative cardiac complications are common, appropriate diagnosis with TTE and TEE has benefited surgeons. TEE-guided reexploration aids surgeons in decision-making and strategic approaches. Failure to diagnose such complications in asymptomatic patients can ultimately complicate the procedure. Henceforth, sonographers must be skilled in the detection and identification of unusual complications to guide redo interventions. Such an approach minimizes mortality, redo procedures, and avoids CPB hence reducing long-term prognosis and outcomes with valve replacement.

**Abbreviations**
ACC: American College of Cardiology
AHA: American Heart Association
AV: Atrioventricular
CHF: Congestive heart failure
CPB: Cardiopulmonary bypass
CT: Computed tomography
2D: Two-dimensional
DVT: Deep vein thrombosis
ECG: Electrocardiogram
EROA: Effective regurgitant orifice area
ICS: Intercostal space
INR: International normalized ratio
IVC: Inferior venacava
LA: Left atria
LV: Left ventricle
LVS3: Third heart sound
LVS4: Fourth heart sound
MR: Mitral regurgitation
MVP: Mitral valve prolapse
NYHA: New York Heart Association
PND: Paroxysmal nocturnal dyspnoea
PE: Pulmonary embolism
PVT: Prosthetic valve thrombosis
RA: Right atria
RV: Right ventricle
SVC: Superior Venacava
S1: First heart Sound
S2: Second Heart sound
TEE: Transesophageal echocardiography
TR: Tricuspid regurgitation
TTE: Transthoracic echocardiography
V1: Precordial lead one
VPC: Ventricular premature complex

## Introduction

Immediate postoperative complications are part and parcel of surgical valve replacement. Commonly witnessed complications include hematoma, bleeding, valve dehiscence, paravalvular leak, and acute prosthetic valve thrombosis. With the data available from published articles, the rate of all valve-related complications was 0.7 to 3.5% per patient annually. ^1^ The pathology involved is multifactorial, often involving blood vessel injury leading to bleeding and hematoma formation. Although postoperative complications are quite evident, we report an unusual case of incidental diagnosis of a cardiac mass in an asymptomatic and hemodynamically stable patient postoperatively, requiring non-invasive imaging in immediate surgical action.

## Case report

A woman in her 50s presented with chief complaints of breathlessness for one year and associated palpitations for the past three months. On evaluation, breathlessness was sudden in onset and progressively worsened from NYHA class II to III. The patient had a history of PND and a known case of rheumatic heart disease (duration unclear) that was managed conservatively. There was no prior history of orthopnea. However, she did report experiencing exertional palpitations characterized by sudden onset and relief upon resting. No family history of cardiac illnesses was documented. On examination, her blood pressure and pulse rate were 130/78 mmHg and 90/min, respectively. Physical examination showed the apex to be outwards and shifted to the lower 6 ^th^ ICS 1 cm lateral to the mid-clavicular line, hyperdynamic in character, and well localized, suggestive of an ‘LV’ type apex. On auscultation, a non-ejection click was heard at the apex with soft S1 and a wide split S2. LVS3 and LVS4 were absent. A mid-systolic murmur of grade 3/6 was heard at the apex immediately after the click, with radiation to the mid axillary line.

### Investigations

**Figure 1.**
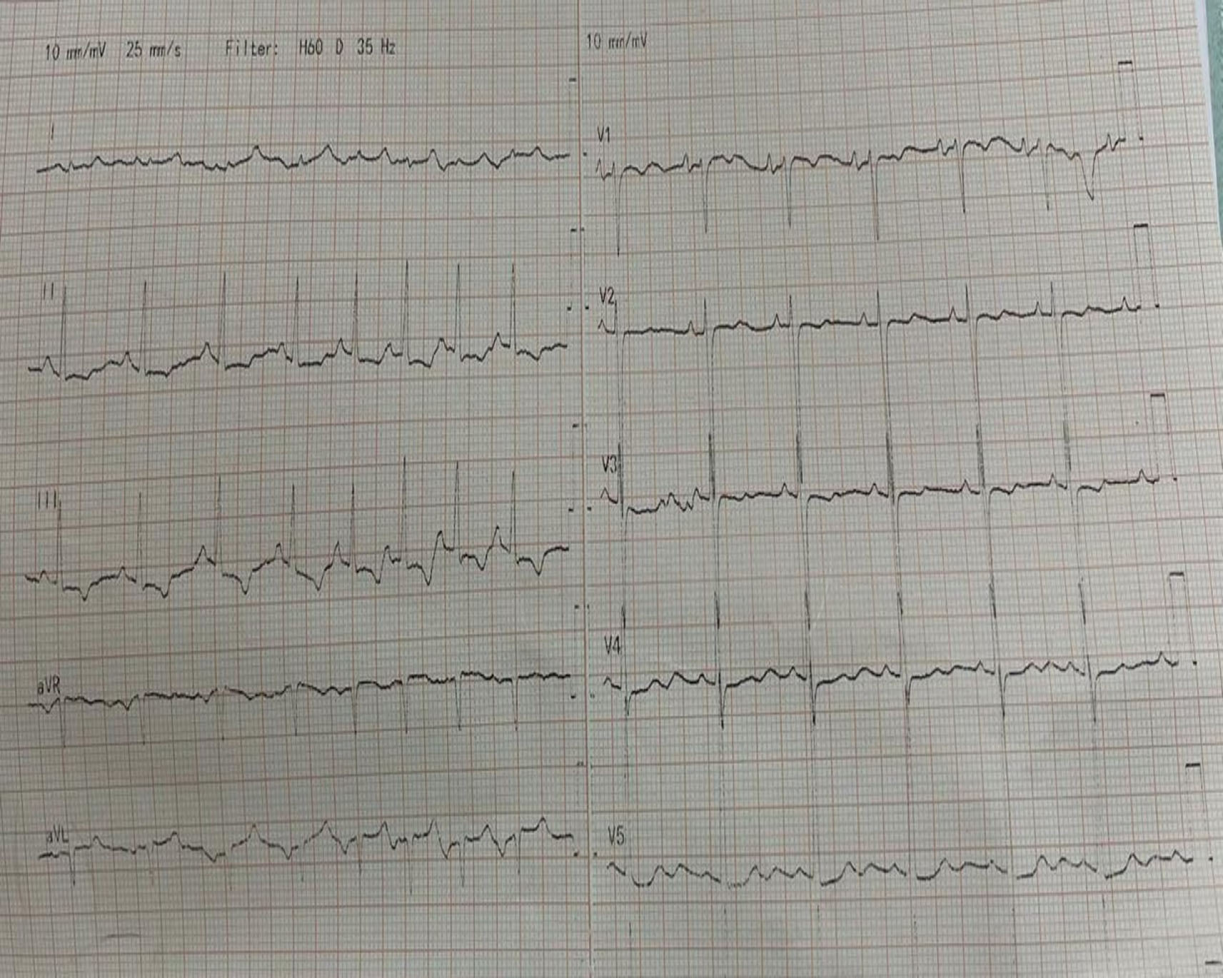
Standard 12-lead ECG demonstrating findings of sinus arrhythmia and biatrial enlargement.

**Figure 2.**
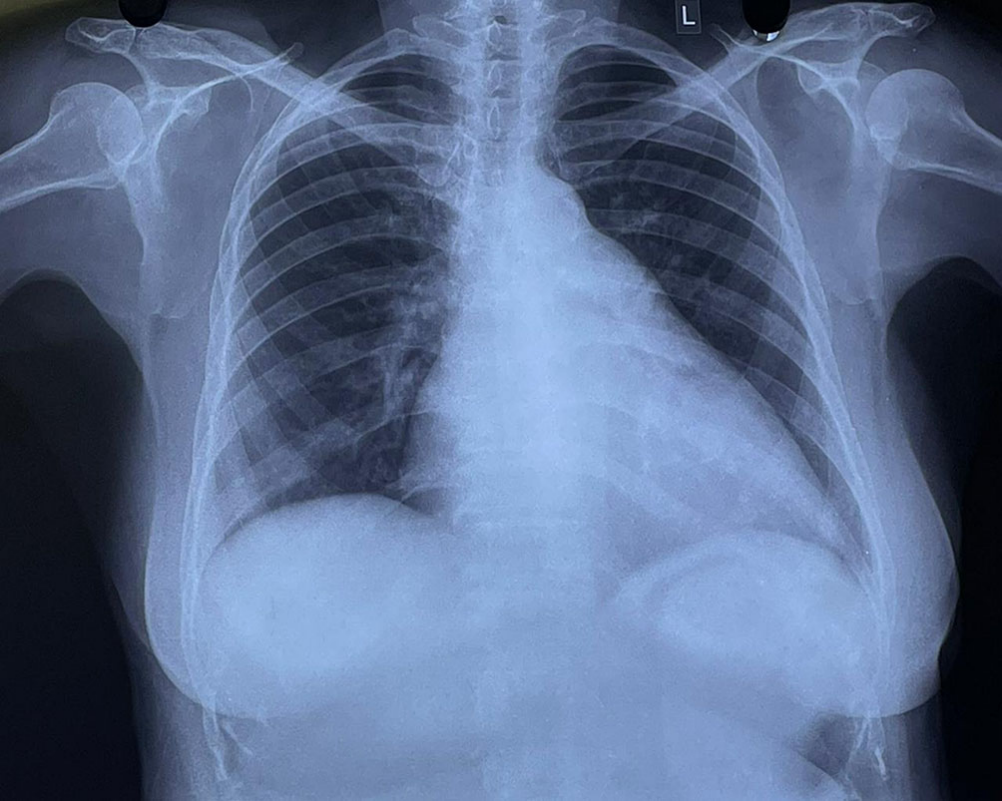
Chest radiograph demonstrating cardiomegaly, straightening of the left heart border and double density shadow.

A standard 12-lead ECG was performed which showed sinus arrhythmia with a notched P wave in the inferior leads and a prominent biphasic P wave in lead V1, suggestive of biatrial enlargement ( Figure 1). Chest radiography revealed cardiomegaly with a double-density shadow and LV enlargement. These findings suggested a left heart aetiology ( Figure 2).

**Figure 3.**
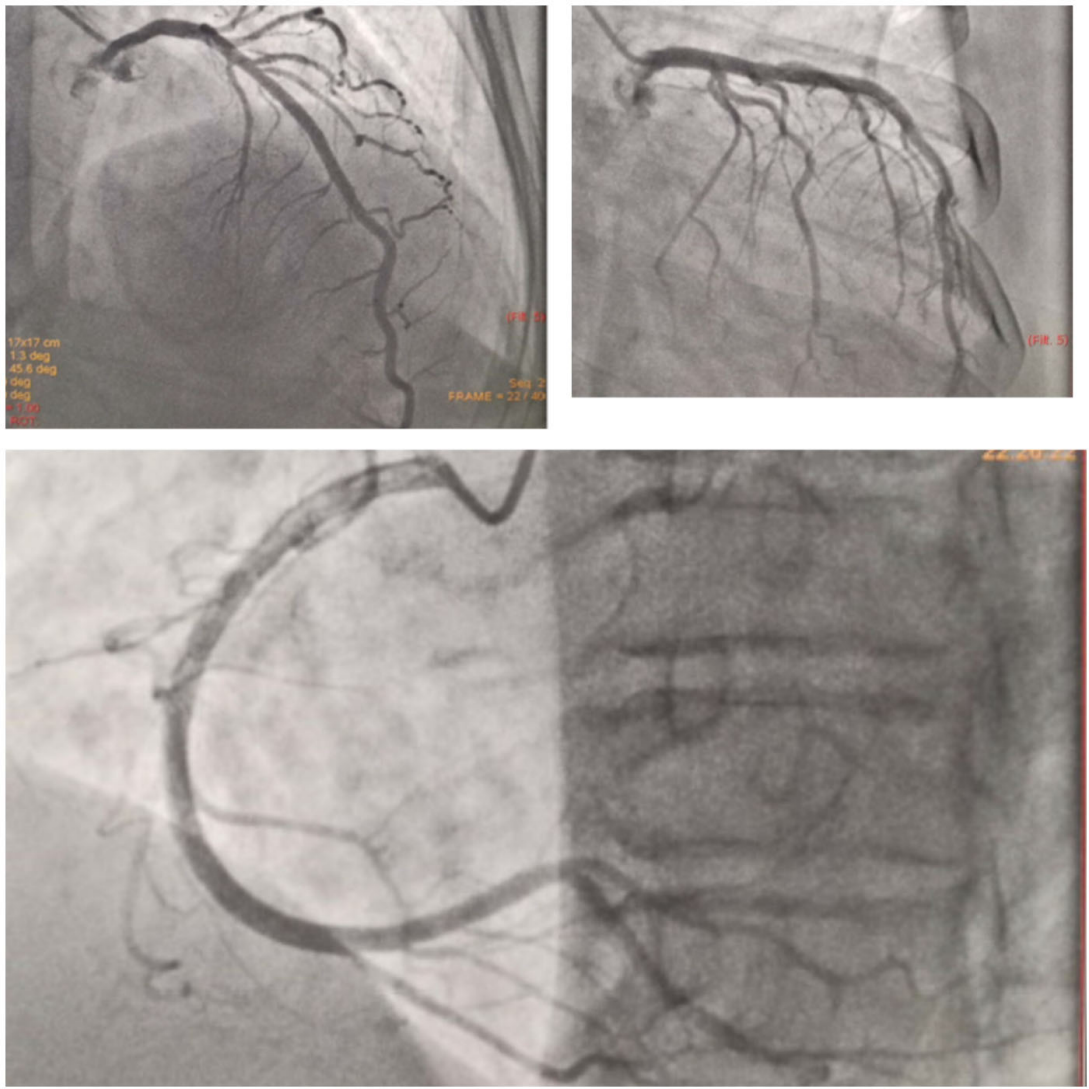
Diagnostic coronary angiogram showing normal epicardial coronary arteries.

2D transthoracic echocardiography was performed one day before the planned procedure. The findings showed mitral valve prolapse (bileaflet) with eccentric MR jet (EROA 0.43 cm ^2^, regurgitant volume = 91 ml), dilated LA, and borderline dilated LV with preserved LV function. The RV function was preserved with mild TR. Absence of pericardial effusion/thrombus was documented during the preoperative echocardiography. 2D echocardiography confirmed severe mitral regurgitation requiring surgery as per the current ACC/AHA class I recommendation and was referred to a cardiothoracic surgeon for further management, wherein she was advised to undergo mitral valve replacement. To rule out coronary artery disease, a diagnostic coronary angiography was performed which revealed normal epicardial arteries ( Figure 3).

Preoperative investigations included routine blood tests, thyroid function tests, serology, cardiac enzymes, urine analysis, and serum electrolytes. The procedure was planned following all the above-mentioned investigated parameters being within their reference limits. All the investigated parameters were within the reference limits, and the procedure was planned accordingly. The procedure was executed with aseptic precautions and general anesthesia. A median sternotomy was performed, where the pericardium was opened and cradled. The patient was adequately heparinized and constantly monitored for the coagulation parameters. Routine aortic and bicaval cannulation of the SVC, IVC, LA was carried out and antegrade cardioplegic cannula was carried out besides initiating CPB. Aorta was cross-clamped and root cardioplegia was administered to arrest the heart. The left atrium was opened, followed by excision and replacement of mitral valve with a 31 M TTK Chitra Heart Valve using a 2/0 pledgetted suture. The valve was checked for its functionality using transoesophageal echocardiography. The left atrium was closed in two layers with 3/0 prolene. Routine deaeration and cross-clamping were performed, and the bypass was smoothly weened off. Protamine was administered and routine decannulation was performed followed by placement of RV pacing wire along with mediastinal drain tubes. On achieving hemostasis, the sternum was closed with steel wires, and the chest was closed in layers.

**Figure 4.**
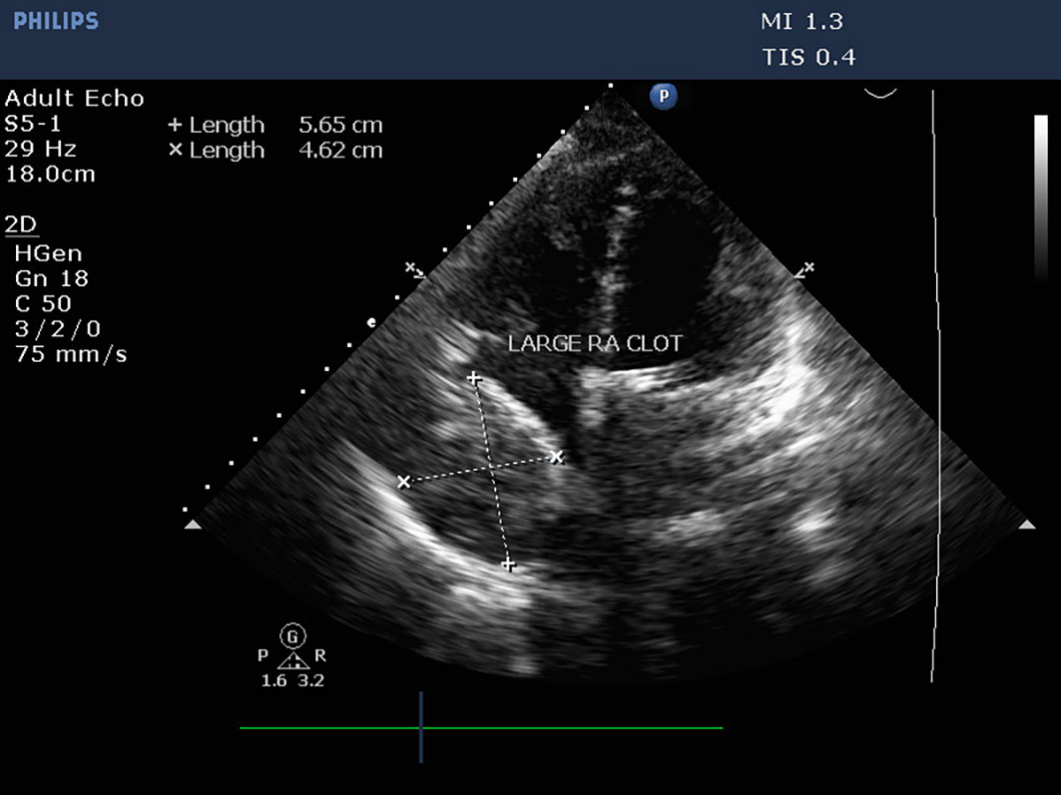
Transthoracic echocardiography demonstrating a large mass occupying the RA cavity after MVR.

The patient was transferred to the intensive care unit for postoperative care. The vital signs were stable, and the patient received inotropic support. Postoperative echocardiography could not be performed because of an insufficient acoustic window soon after the patient was received. However, TTE was performed within 24 hours to assess prosthetic valve function. Valve functioning appeared optimal, with a trivial transvalvular leak. The forward flow gradients were acceptable (5/3 mmHg) with adequate biventricular function. Postoperative echo remarkably showed a large mass measuring 5.6 cm*4.6 cm extensively occupying the RA cavity when examined from the apical 4-chamber (Video 1) and subcostal 4-chamber views (Video 2). The subcostal IVC long axis showed an IVC within normal limits and adequate collapsibility (Video 3). No flow obstruction was noted across the tricuspid valve or SVC ( Figure 4) (Video 4).

**Figure 5.**
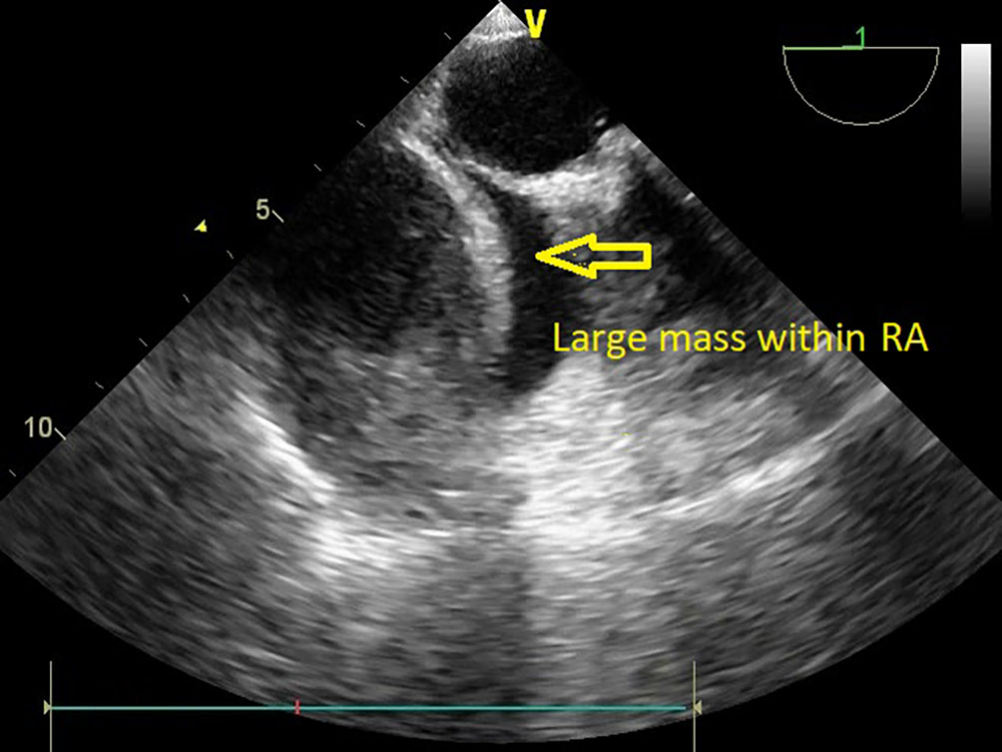
Postoperative TEE performed to identify the extent and location of the mass in RA.

Although the patient was adequately anticoagulated post procedure, transoesophageal echocardiography confirmed the presence and extension of a mass (Video 5) which intruded the RA cavity. An organized echogenic mass, homogenous in appearance, was observed along the lateral wall of the RA with regular borders ( Figure 5) (Video 6).

### Differential diagnosis

Mitral valve replacement was performed using the traditional approach of a vertical left atriotomy. Susceptibility to thrombus formation can be anticipated due to the presence of a prosthetic valve or sluggish flow in the setting of CHF or cardiomyopathy, which was not observed. The likelihood of thrombus formation within the right atrium was considered the to be least likely based on the echocardiographic diagnosis, since the patient had no provoking factors, as listed above. Intraoperative echocardiography was performed to assess cardiac function before proceeding with surgery, which revealed the absence of clots within the right atrium and major venous channels. Although the atria were dilated, no spontaneous contrast was observed within the chambers, which could be a precursor to thrombus formation.

Normal anatomical variants include prominent thymus glands situated in the superior and anterior mediastinum. It intervenes between the sternum in the front and the pericardium located between the right atria. The thymus progressively increases in size during puberty and undergoes involution at a later age. From the surgeon’s point of view, the thymus was unnoticeable during the examination and was likely to be mistaken for a mass on echocardiography. No history of radiation and chemotherapy or hypercoagulable state/DVT/PE was documented.

Postoperative radiography revealed the absence of mediastinal widening with a well-functioning prosthesis and acceptable transvalvular gradients. TEE exhibited good prosthetic function in the absence of a thrombus. The above-listed conditions were not observed and thus omitted before concluding the possible causes of complications. Various studies have identified common immediate postoperative complications, including active bleeding, PVT, wound infection, valve dehiscence causing paravalvular leak, and mediastinal haemorrhage that required reoperation. Other complications include apical extra pleural hematomas, which were identified on chest radiographs as mediastinal widening with increased mediastinal tube drainage. ^2^ Therefore, we believe that a possible complication favouring intracardiac or extracardiac mass requires reexploration under the guidance of TEE.

### Transoesophageal echocardiography

**Figure 6.**
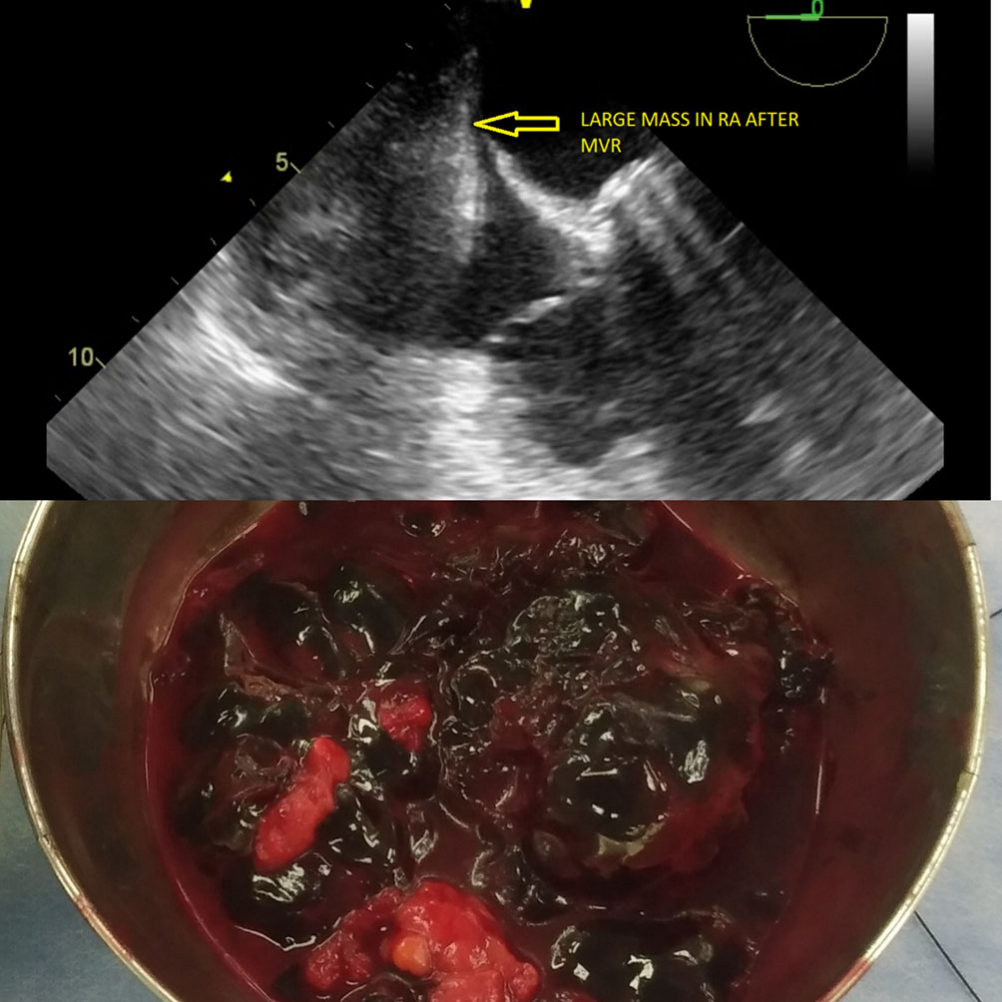
Intraoperative reexploration under TEE guidance showing multiple clots.

On TEE, prosthetic valve function was good (Video 7), with trivial transvalvular leakage and a non-thrombosed valve with a large mass occupying the RA cavity ( Figure 6). This led surgeons to decide whether to re-explore the heart or chest only. TEE was performed on a table to assist the surgeon in deciding on a strategic approach for mass excision.

### Treatment

**Figure 7.**
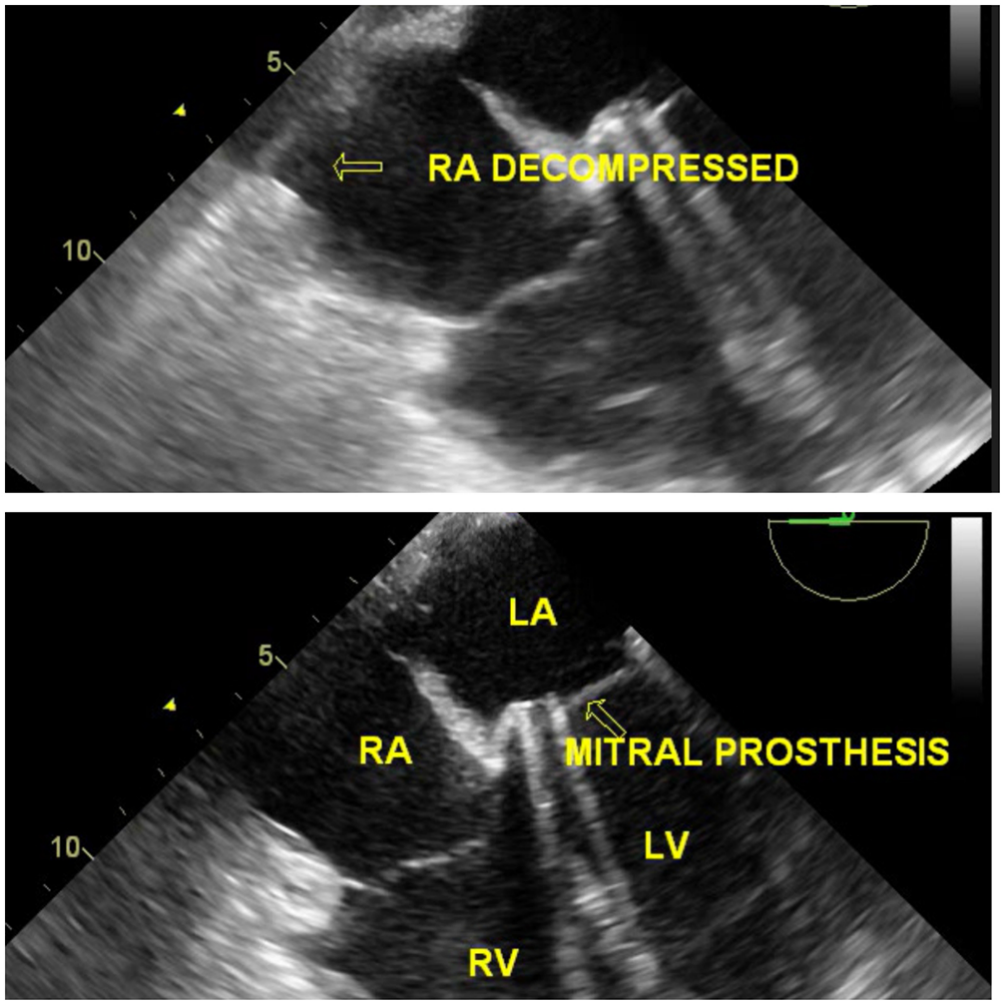
TEE demonstrating a decompressed RA cavity after clot excision with a mitral prosthesis in situ.

**Figure 8.**
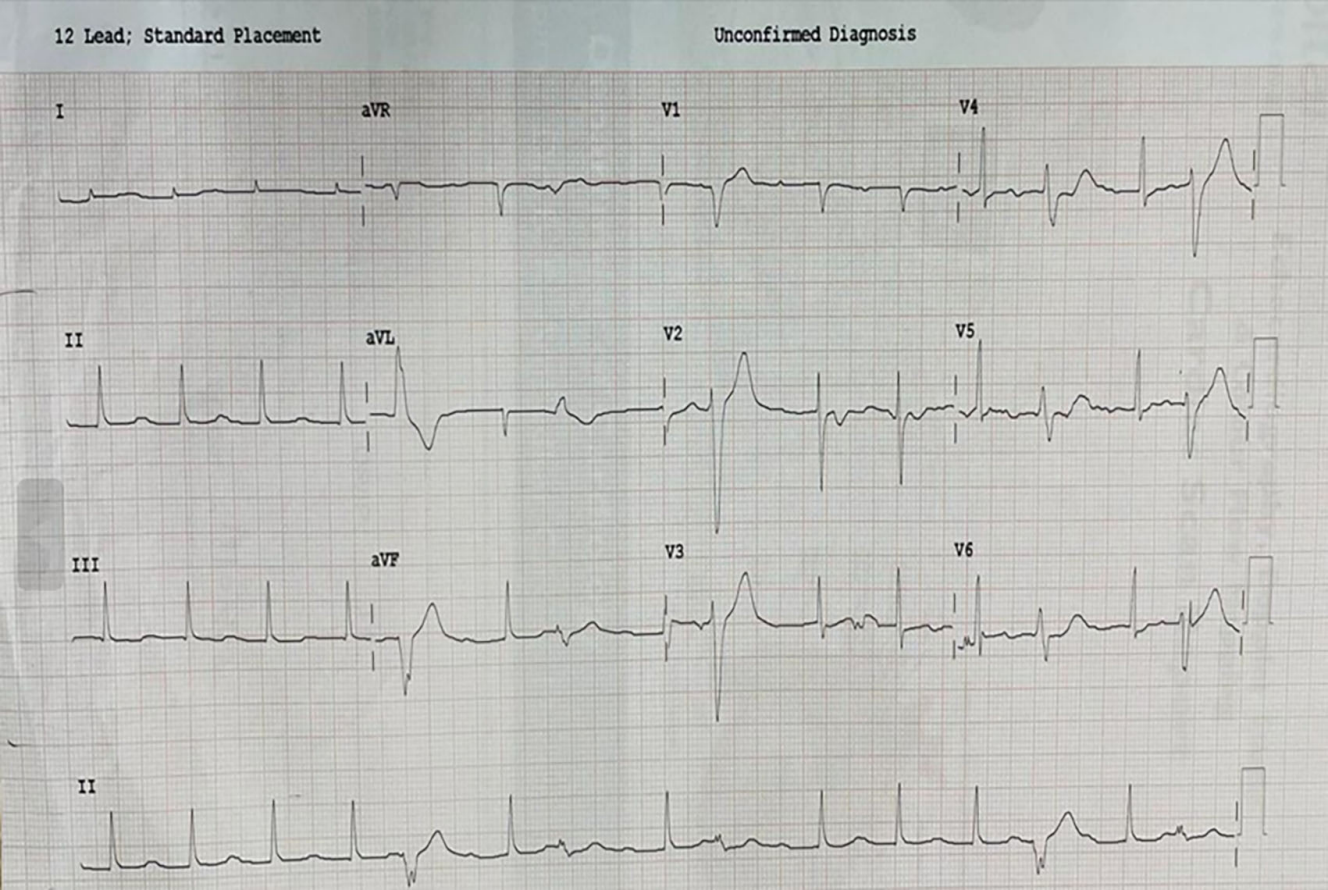
Postoperative 12-lead ECG showing 1 ^st^ degree AV block and frequent VPC’s.

The diagnosis required immediate management and surgical excision under TEE imaging, with or without exploration of the right atrium. Reexploration was planned under TEE guidance and CPB as a standby if the location was intracardiac. The procedure was performed under general anaesthesia. The sternal wires were then opened and retracted. Multiple organized clots were noted in excess, over the right atrium compressing it externally, consequently placing them intrapericardially (extracardiac) ( Figure 6). The clots were excised and had no signs of active bleeding. Post reexploration, TEE did not disclose any intracardiac mass (Video 8), and the RA was decompressed; hence, the patient did not undergo cardiopulmonary bypass ( Figure 7) (Video 9). Prosthetic valve function was good after excision of the clot (Video 10). Postoperative ECG showed 1 ^st^-degree AV block and frequent VPCs ( Figure 8).

**Figure 9.**
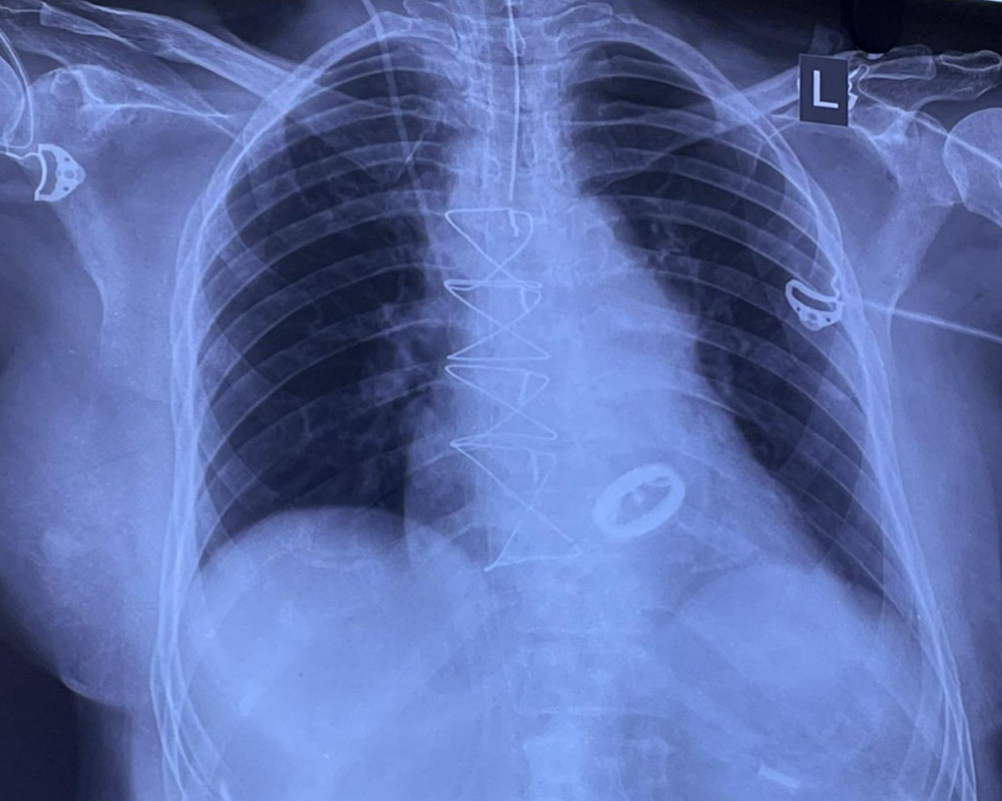
Postoperative chest X-ray showing a mitral prosthesis in situ with intact sternal rings.

Chest radiography was performed after re-exploration that showed intact sternal rings, with well seated mitral prosthesis ( Figure 9). The patient was stable and transferred to the intensive care unit. Repeat blood investigations were performed, and the output was monitored. The serum creatine and electrolyte levels were within the reference limits. She was advised to undergo physiotherapy rehabilitation as supportive care and was discharged a week later.

### Outcome and follow-up

The patient was stable on discharge and was asked to monitor the INR and revisit the physician after noticing any signs of bleeding or worsening symptoms. Postoperative echocardiography showed a well-functioning prosthesis and resolution of the clot on the RA side. The patient was symptomatically better (NYHA class I) during the first follow-up at 15 days post-discharge. Chest radiography showed prosthesis in situ with no signs of pericardial or pleural effusion. Currently, she is under regular follow-up and is being screened for prosthetic valve function and symptoms.

## Discussion

Postoperative valve-related complications commonly include site and mediastinal bleeding, prosthetic valve dysfunction, prosthetic valve endocarditis, and thromboembolic events. ^3^ Complications are elements of valvular surgery that require intense follow-up; hence, echocardiography plays a vital role in the detection of early complications. However, British guidelines recommend echocardiographic imaging soon after implantation to detect early valve deterioration and plan accordingly on the timing for redo intervention. ^4^ From the point of care, repeat echocardiography is indicated at a frequency that depends on the underlying pathology in order to assess LV performance and pericardial effusion postoperatively.

Echocardiography is indicated if there is a suspicion based on the new onset of symptoms that commonly include breathlessness, fever, or clinical findings with a new onset of murmur. ^4^ Patients undergoing valve replacement require careful postoperative follow-up. Unlike the abovementioned complications, this case had an unusual presentation of extracardiac/intrapericardial hematoma that extrinsically compressed the right atrium with no evidence of pericardial tamponade, an immediate postoperative complication.

A similar case reported intrapericardial hematoma presenting with isolated right atrial tamponade, wherein the diagnosis was confirmed by computed tomography (CT) followed by transthoracic echocardiography. ^5^

Studies state that, a prior history of cerebrovascular events is a risk factor for thromboembolic episodes or bleeding complications. ^6^^–^^8^ Moreover, studies have reported that significant mediastinal bleeding requiring exploration is not related to preoperative coagulation factors, anticoagulant use, or total cardiopulmonary bypass time. ^9^ The current patient experienced bleeding within 24 h after the surgery, which was evident by the collection of blood in the mediastinal drains. This is a routine bleeding observed in most cardiac surgery patients, as heparin was used during the procedure. Postoperative complications are likely to be missed if they are located outside of the imaging plane. ^10^

The current study showed that, hematoma surrounding the right atrium as a result of bleeding due to blood vessel injury was difficult to distinguish as intrapericardial/extracardiac hematoma from the djacent tissues on TTE. Although TEE imaging is considered standard imaging during cardiac procedures, its utility has yielded greater assistance for surgeons in tracing hematoma and its extent, limiting procedural time, considering suitable decisions, and looking for any residue post exploration. The necessity of TEE is imperative for further management in this case. Although studies state TEE as point of care peri-and post-operative, it aids surgeons in excising hematoma on table, thereby limiting the CPB time and minimizing bleeding as a result of anticoagulation.

Hence, a thorough echocardiographic examination through multiple windows postoperatively is essential, as mediastinal bleeding involves multifactorial mechanisms. This case report provides insights into the importance of TEE and its utility both operatively and postoperatively, aiding surgeons in resolving this dilemma and considering appropriate management. we thereby conclude that TEE-guided reexploration has enormously directed surgeons in making notable decisions.

### Patient perspective

“Her initial diagnosis of the disease was made at a tertiary care hospital. We explained the disease severity and prognosis, if not treated. Her symptoms worsened, limiting her daily activities. Initially, we were concerned about the major surgery, but post counseling, we decided to move ahead. We were explained about the procedural complications by the surgeon. Postsurgical nursing care was helpful in her early recovery. The initial days after discharge were difficult, but with constant family support, she was able to adjust to the environment and perform routine activities independently. We are very thankful to the hospitality and care provided by the health care staffs throughout her stay”.

Details provided by patient’s son.

#### Ethics and consent

Ethical approval was not required as it is case report. Written informed consent was acquired from the patient’s representative, with approval for waiver granted by the ethics committee. The patient’s guardian has granted consent for publication of the case details while ensuring participant anonymity. The patient has also agreed to the submission of the case report to the journal.

## Data Availability

No data were associated with this article. Figshare: Case Report: Post-Operative Mitral Valve Replacement Complicating with a Large Cardiac Mass and Role of TEE in Decision Making”, figshare data, 10.6084/m9.figshare.25547809.v1. ^11^ This project contains the following underlying data: Date file 1: Case images Date file 2: Media files and Care checklist Data are available under the terms of the Creative Commons Attribution 4.0 International license (CC-BY 4.0).
