# Case Report: Post-operative mitral valve replacement complicating with a large cardiac mass and the role of TEE in Imaging

**DOI:** 10.12688/f1000research.145007.2

**Published:** 2024-10-01

**Authors:** Narasimha Pai D, Chaithra Nayak, Padmanabh Kamath, Syed Waleem Pasha, Deepa Noronha

**Affiliations:** 1 Department of Cardiology, Kasturba Medical College, Mangalore, Manipal Academy of Higher Education, Karnataka, Manipal, 576104, India

**Keywords:** Prosthetic mitral valve, Echocardiography, Reexploration, Cardiac mass, Complication

## Abstract

**Background:**

Postoperative complications are an integral part of valve surgery. Common complications include hematomas, bleeding, valve dehiscence, paravalvular leak, and acute PV thrombosis. With the available data from published articles, the rate of all valve-related complications is 0.7 to 3.5% per patient annually. [1] The pathology involved is multifactorial, often blood vessel injury leading to bleeding and hematoma. Although postoperative complications are evident, incidental diagnosis of a cardiac mass in an asymptomatic and hemodynamically stable patient postoperatively is crucial, requiring non-invasive imaging for immediate surgical action.

**Case presentation:**

A woman in her 50s presented with chief complaints of worsening dyspnoea with suddenonset and chest pain. Clinical findings showed apex shifted downward and outward, wide split S2, and a mid systolic murmur radiating to the mid axillary line. Twelve-lead ECG showed LA enlargement, that aligned with X-ray findings. 2D Echocardiography revealed MVP with severe MR and a dilated LV. The patient underwent successful mitral valve replacement as per ACC/AHA class I recommendation. However, postoperative TTE showed a remarkably large mass measuring 5.6 cm*4.6 cm in the RA. Reexploration was performed, followed by mass excision. Plenty of organized clots were seen compressing the RA. TEE showed no evidence of mass. Following stabilization,the patient was discharged considering optimal INR values and prosthetic valve function assessed by echocardiography. The patient’s symptoms improved during the first follow-up.

**Conclusion:**

Although postoperative cardiac complications are common, appropriate diagnosis with TTE and TEE has benefited surgeons. TEE-guided reexploration aids surgeons in decision-making and strategic approaches. Failure to diagnose such complications in asymptomatic patients can ultimately complicate the procedure. Henceforth, sonographers must be skilled in the detection and identification of unusual complications to guide redo interventions. Such an approach minimizes mortality, redo procedures, and avoids CPB hence reducing long-term prognosis and outcomes with valve replacement.

**Abbreviations**
ACC: American College of Cardiology
AHA: American Heart Association
AV: Atrioventricular
CHF: Congestive heart failure
CPB: Cardiopulmonary bypass
CT: Computed tomography
2D: Two-dimensional
DVT: Deep vein thrombosis
ECG: Electrocardiogram
EROA: Effective regurgitant orifice area
ICS: Intercostal space
INR: International normalized ratio
IVC: Inferior venacava
LA: Left atria
LV: Left ventricle
LVS3: Third heart sound
LVS4: Fourth heart sound
MR: Mitral regurgitation
MVP: Mitral valve prolapse
NYHA: New York Heart Association
PND: Paroxysmal nocturnal dyspnoea
PE: Pulmonary embolism
PVT: Prosthetic valve thrombosis
RA: Right atria
RV: Right ventricle
SVC: Superior Venacava
S1: First heart Sound
S2: Second Heart sound
TEE: Transesophageal echocardiography
TR: Tricuspid regurgitation
TTE: Transthoracic echocardiography
V1: Precordial lead one
VPC: Ventricular premature complex

## Introduction

The formation of intrapericardial hematomas occurs due to rapid or slow blood accumulation, often accompanied by cardiac surgery. Although such complications are witnessed rarely, they can cause a significant impact on cardiac hemodynamics from milder to serious life-threatening conditions, in-hospital mortality, and prolonged hospital stay. ^1^ Identification of such hematomas by cardiac imaging is necessary for evacuation once the diagnosis is established. It is important to stress that postoperative complications are an integral part of valve surgery and include common complications like hematomas, bleeding, valve dehiscence, paravalvular leak, and acute PV thrombosis. With the available data from published articles, the rate of all valve-related complications is 0.7 to 3.5% per patient annually. ^2^ The pathology involved is multifactorial, often blood vessel injury leading to bleeding and hematoma. Although postoperative complications are evident, incidental diagnosis of a cardiac mass in an asymptomatic and hemodynamically stable patient postoperatively is crucial, requiring reexploration and surgical mass excision.

## Case report

### Case presentation

***Patient information***

A woman in her 50s presented with chief complaints of breathlessness for one year and associated palpitations for the past three months. On evaluation, breathlessness was sudden in onset and progressively worsened from NYHA class II to III. The patient reported with a history of PND and rheumatic heart disease (duration unclear) that was managed conservatively. There was no documented history of orthopnoea. She complained of palpitations that was exertional, with sudden onset and relief during rest. There was no recorded history of cardiac disease in the family.

***Clinical examination***

On examination, her blood pressure was 130/78 mmHg and pulse rate was 90/min. The physical examination revelaed the apex beat to be displaced outward and shifted to the lower 6 ^th^ intercostal space, 1 cm lateral to the mid-clavicualr line. It was hyperdynamic and well localized, suggestive of left ventricular (LV) type apex. Auscultation revelaed a non-ejection click at the apex. The first heart sound (S1) was soft, and the second heart sound (S2) was widely split. LV S3 or S4 sounds were absent. A grade 3/6 mid-systolic murmur, occurring immediately after the click, was heard at the apex with radiation to the mid-axillary line.

### Investigations

***12 lead electrocardiography***

**Figure 1.**
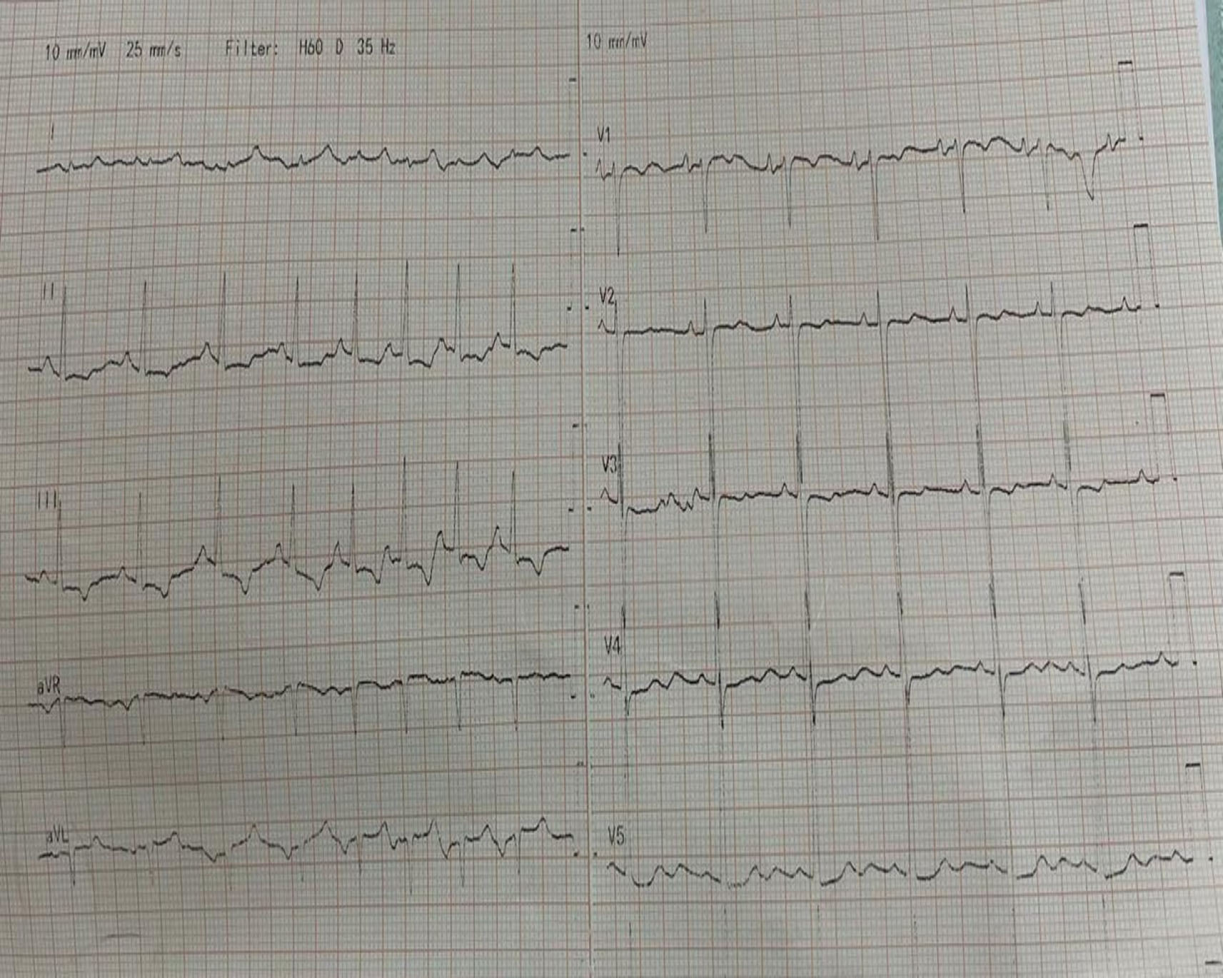
Standard 12-lead ECG demonstrating findings of sinus arrhythmia and biatrial enlargement.

**Figure 2.**
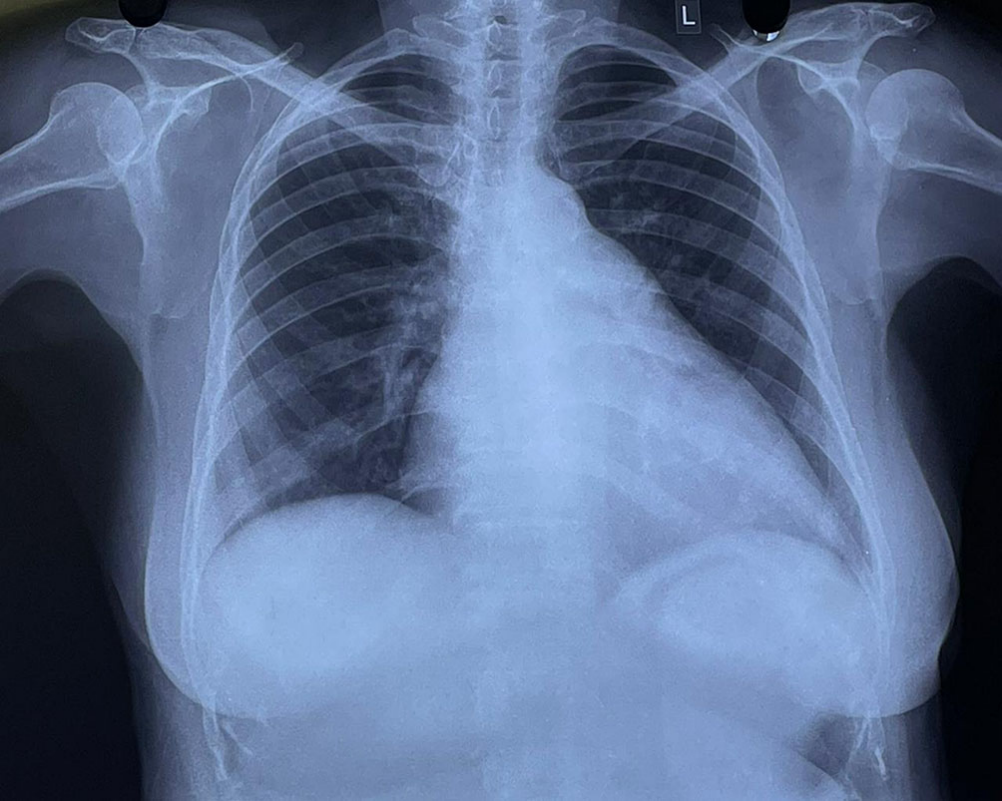
Chest radiograph demonstrating cardiomegaly, straightening of the left heart border and double density shadow.

A standard 12-lead ECG was conducted, revealing sinus arrhythmia with a notched P wave in the inferior leads and a prominent biphasic P wave in lead V1, indicating possible biatrial enlargement ( Figure 1). Chest X-ray showed cardiomegaly with a double-density shadow and left ventricular enlargement. These findings pointed to a left-sided heart etiology ( Figure 2).

***2D Echocardiography***

**Figure 3.**
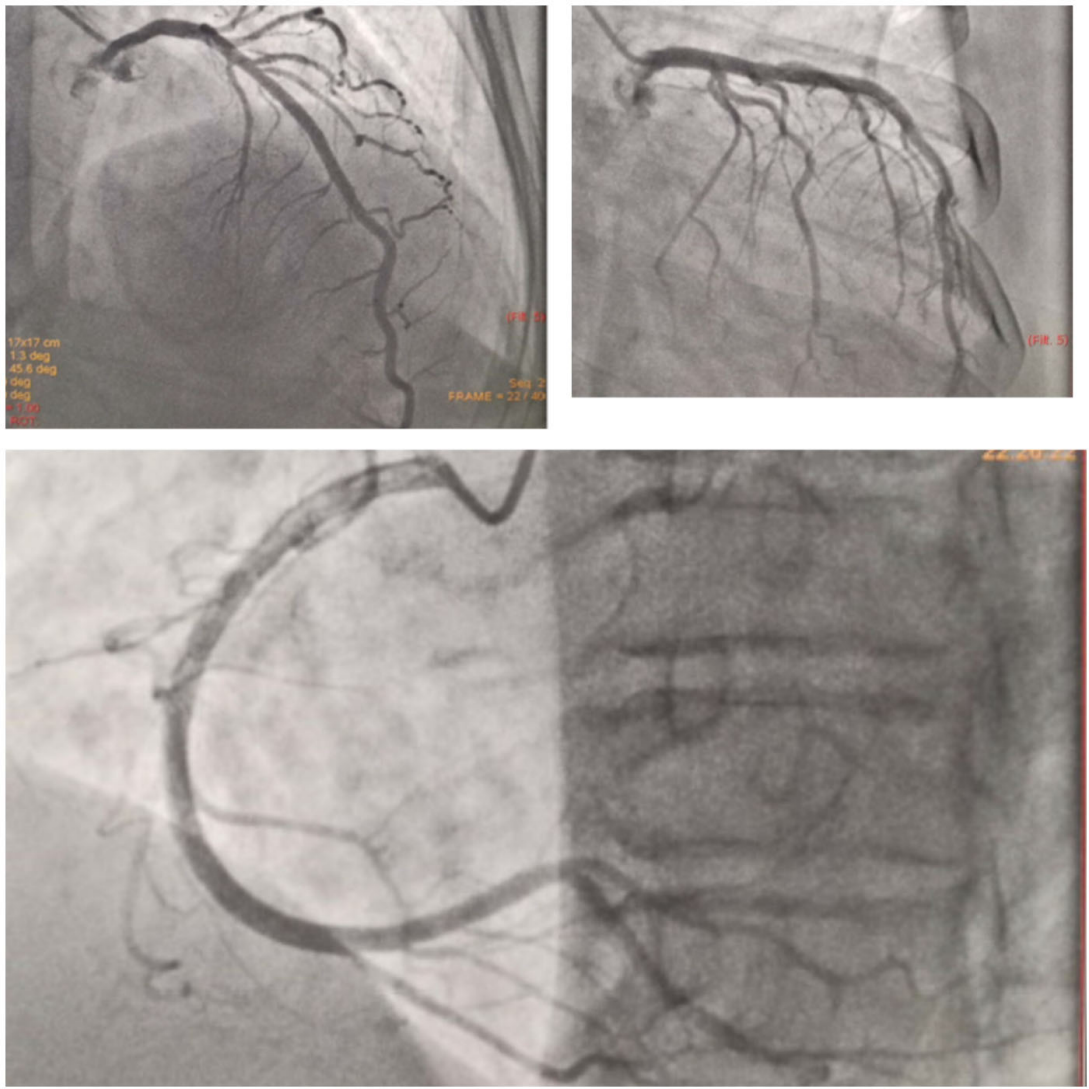
Diagnostic coronary angiogram showing normal epicardial coronary arteries.

2D transthoracic echocardiography was performed one day before the planned procedure. The findings showed mitral valve prolapse (bileaflet) with eccentric MR jet (EROA 0.43 cm ^2^, regurgitant volume = 91 ml), dilated LA, and borderline dilated LV with preserved LV function. Right heart function was preserved with mild tricuspid insufficiency. Pericardial effusion/thrombus was absent and therefore documented during the preoperative echocardiography. 2D echocardiography confirmed severe mitral regurgitation requiring surgery as per the current ACC/AHA class I recommendation and was referred to a cardiothoracic surgeon for further management. She was advised to undergo mitral valve replacement. A diagnostic coronary angiogram, was performed to rule out coronary artery disease. However, coronary angiography revealed normal epicardial arteries ( Figure 3).

***Further clinical findings***

Preoperative assessments included routine blood tests, thyroid function tests, serology, cardiac enzymes, urinalysis, and serum electrolytes. All results were within normal reference ranges, and the procedure was planned based on these findings.

### Surgical intervention

The procedure was executed with aseptic precautions and general anesthesia. A median sternotomy was performed, where the pericardium was opened and cradled. The patient was adequately heparinized and constantly monitored for the coagulation parameters. Routine aortic and bicaval cannulation of the SVC, IVC, LA vent, and antegrade cardioplegic cannula was performed, and CPB was initiated. The aorta was cross-clamped and root cardioplegia was administered to arrest the heart. The left atrium was opened, followed by excision and replacement of mitral valve with a 31 M TTK Chitra Heart Valve using a 2/0 pledgetted suture. The valve was checked for its functionality using transoesophageal echocardiography. The left atrium was closed in two layers with 3/0 prolene. Routine deaeration and cross-clamping were performed, and the bypass was smoothly removed. Protamine was administered and routine decannulation was performed followed by placement of RV pacing wire along with mediastinal drain tubes. On achieving hemostasis, the sternum was closed with steel wires, and the chest was closed in layers.

***Postoperative findings***

The patient was transferred to the intensive care unit for postoperative care. She was continuously assessed for vital signs and closely monitored for neurological status and surgical site for active signs of bleeding. Patient was adequately hydrated, and fluid balance was closely monitored. The patient was adequately anticoagulated for DVT prophylaxis. Postoperative echocardiography could not be performed because of an insufficient acoustic window soon after the patient was received.

**Figure 4.**
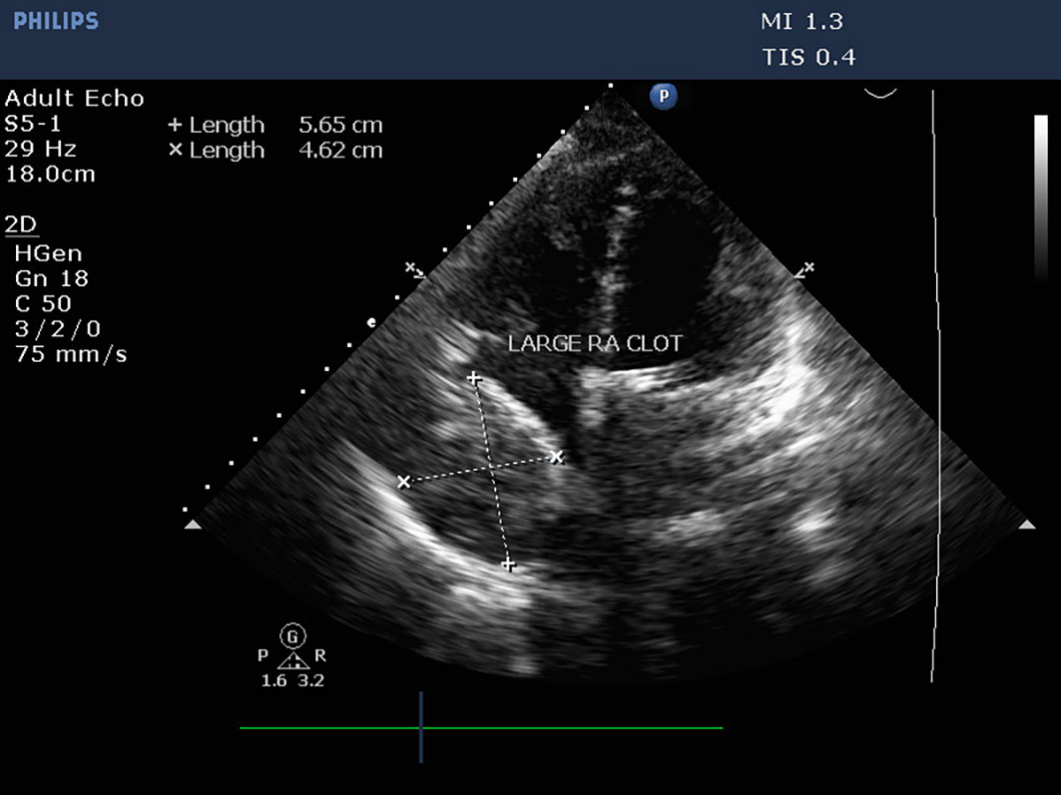
Transthoracic echocardiography demonstrating a large mass occupying the RA cavity after MVR.

However, TTE was performed within 24 hours to assess prosthetic valve function. Valve functioning appeared optimal, with a trivial transvalvular leak. The forward flow gradients were acceptable (5/3 mmHg) with adequate biventricular function. Postoperative echo remarkably showed a large mass measuring 5.6 cm*4.6 cm surrounding RA cavity when examined from the apical 4-chamber (Video 1) and subcostal 4-chamber views (Video 2). The subcostal IVC long axis showed an IVC within normal limits and adequate collapsibility (Video 3). No flow obstruction was noted across the tricuspid valve or SVC ( Figure 4) (Video 4).

***Advanced imaging***

**Figure 5.**
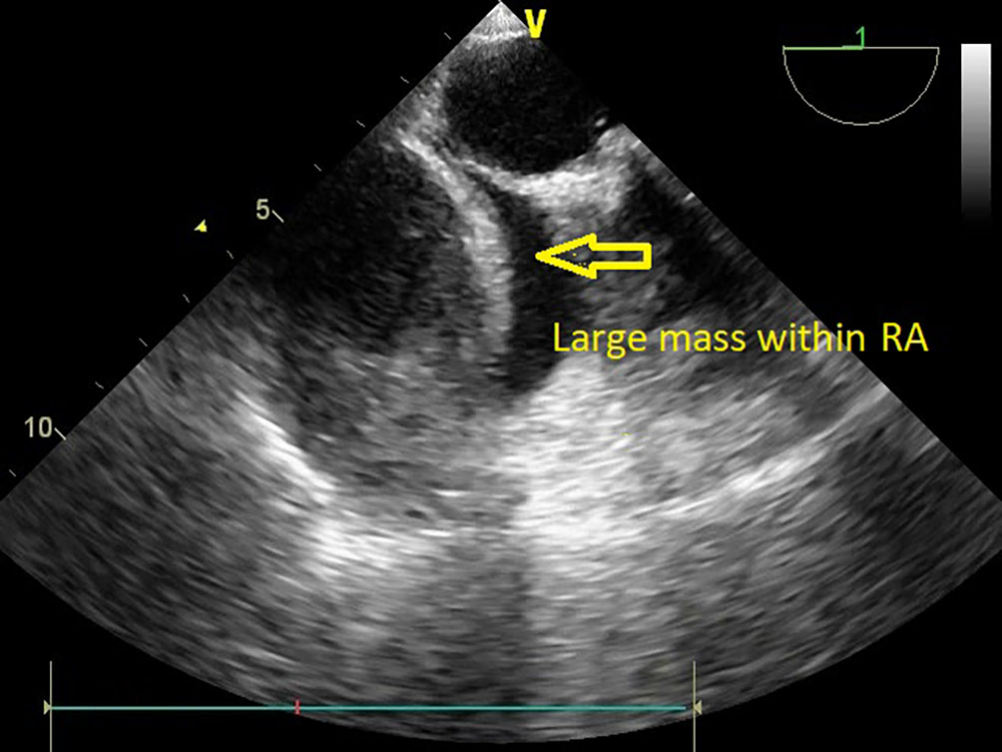
Postoperative TEE performed to identify the extent and location of the mass in RA.

Due to patient characteristics, TTE could not provide detailed information about the mass, its extent, or its impact on adjacent structures, impeding diagnostic findings. Consequently, a TEE was performed to assess the mass’s dimensions and extent and to check for any chamber compression or blood flow obstruction (Video 5). The mass was found to have invaded the RA cavity and appeared as a well-organized, homogeneous echogenic mass along the lateral wall of the RA with regular borders ( Figure 5) (Video 6). However, 2D TEE was insufficient to determine whether the mass was intra- or extracardiac.

## Differential diagnosis

Mitral valve replacement was performed using the traditional approach of a vertical left atriotomy. Thrombus formation was considered unlikely, despite the presence of a prosthetic valve or potential sluggish flow in the context of CHF or cardiomyopathy. The echocardiographic diagnosis indicated a least possibility of thrombus formation in the right atrium considering the patient had no provokable factors.

Intraoperative echocardiography was conducted to assess cardiac function before surgery, revealing no clots within the right atrium or major venous channels. Although the atria were dilated, no spontaneous contrast was observed within the chambers, which could indicate a precursor to thrombus formation.

Normal anatomical variants include prominent thymus glands situated in the superior and anterior mediastinum. It intervenes between the sternum in the front and the pericardium located between the right atria. The thymus progressively increases in size during puberty and undergoes involution at a later age. From the surgeon’s point of view, the thymus was unnoticeable during the examination which could have led to misinterpretation as mass on echocardiography. There was no documented history of radiation or chemotherapy. The patient had no history of hypercoagulable state/DVT/PE.

Postoperative radiography revealed the absence of mediastinal widening with a well-functioning prosthesis and acceptable transvalvular gradients. TEE revealed good prosthetic function. The above-listed conditions were not observed and thus omitted before concluding the possible causes of complications. Various studies have identified common immediate postoperative complications, including active bleeding, PVT, wound infection, valve dehiscence causing paravalvular leak, and mediastinal haemorrhage that required reoperation. Other complications include apical extra pleural hematomas, which were identified on chest radiographs as mediastinal widening with increased mediastinal tube drainage. ^2^ Therefore, we believe that a possible complication would favour intracardiac or extracardiac mass thereby requiring reexploration.

### Intraoperative TEE

**Figure 6.**
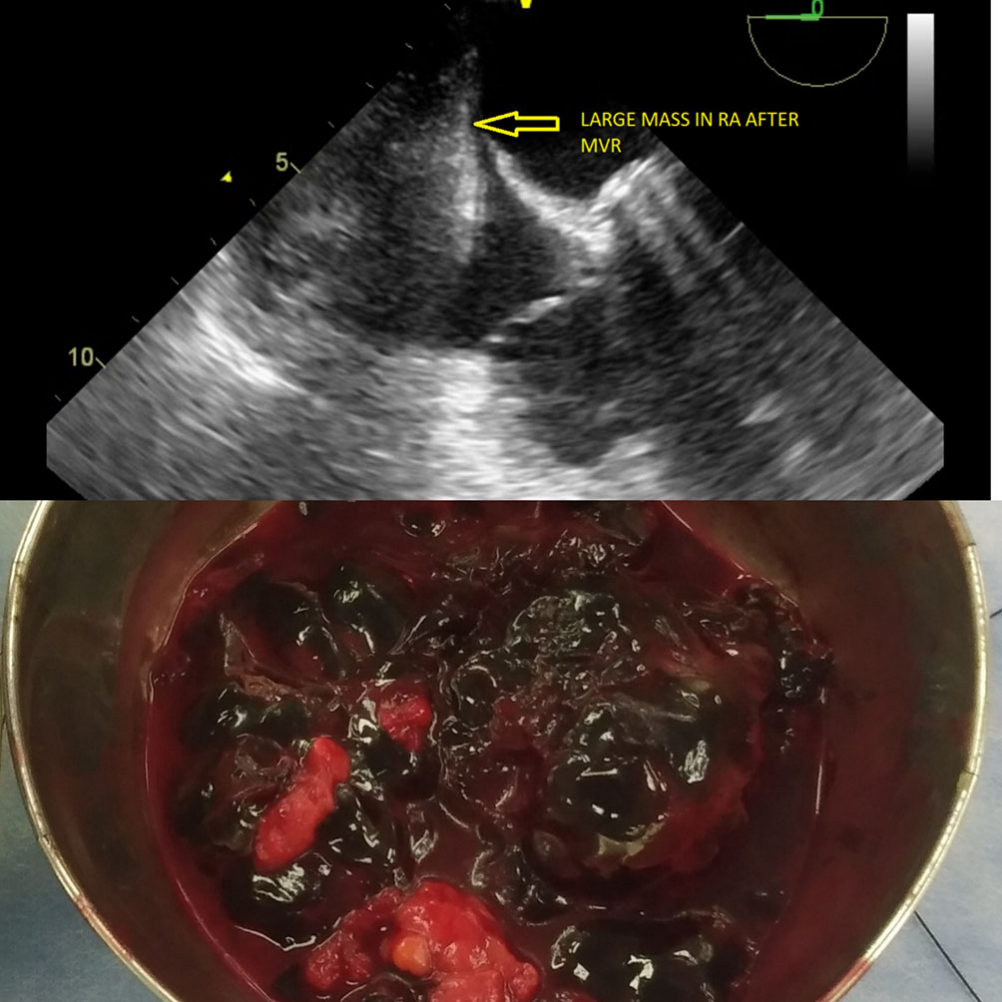
Intraoperative reexploration under TEE guidance showing multiple clots.

A TEE was kept on standby before the initiation of re-exploration. The prosthetic valve function was good (Video 7), with trivial transvalvular leakage and a non-thrombosed valve with a large mass occupying the RA cavity ( Figure 6). However, the location of the mass was indistinguishable as either intracardiac or extracardiac on 2D TEE and failed to prompt the surgeon to re-explore the chest or heart only.

### Treatment

**Figure 7.**
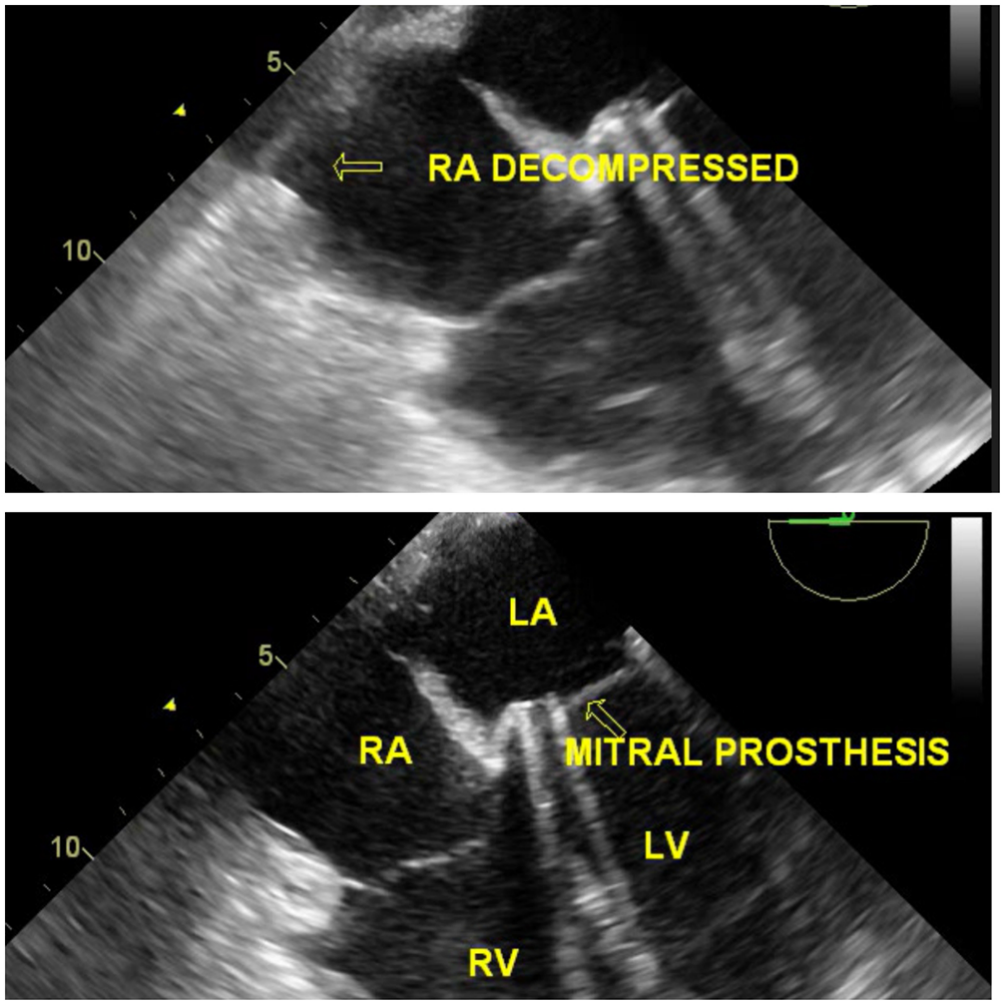
TEE demonstrating a decompressed RA cavity after clot excision with a mitral prosthesis in situ.

**Figure 8.**
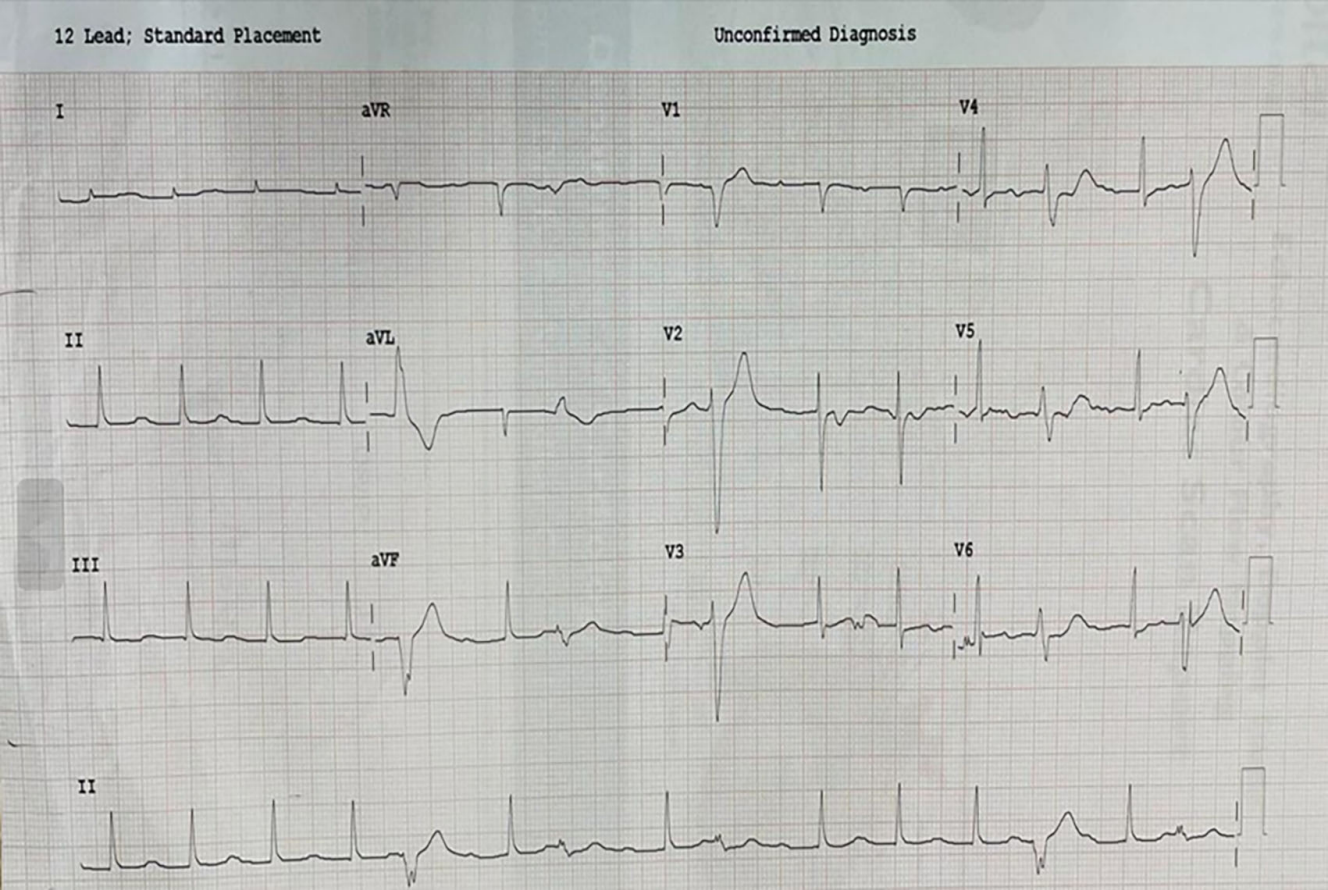
Postoperative 12-lead ECG showing 1 ^st^ degree AV block and frequent VPC’s.

The diagnosis required immediate management and surgical excision under TEE imaging, with or without exploration of the right atrium. Reexploration was planned under TEE guidance and CPB as a standby if the location was intracardiac. The procedure was performed under general anaesthesia. The sternal wires were then opened and retracted. Multiple organized clots were noted in excess, over the right atrium externally and placed intrapericardially (extracardiac) ( Figure 6). The clots were excised and had no signs of active bleeding. Subsequently, post reexploration TEE did not disclose any intracardiac mass (Video 8), and the RA was decompressed; hence, the procedure did not necessitate the use of cardiopulmonary bypass ( Figure 7) (Video 9). Prosthetic valve function satisfactory (Video 10). Postoperative ECG showed 1 ^st^-degree AV block and frequent VPCs ( Figure 8).

**Figure 9.**
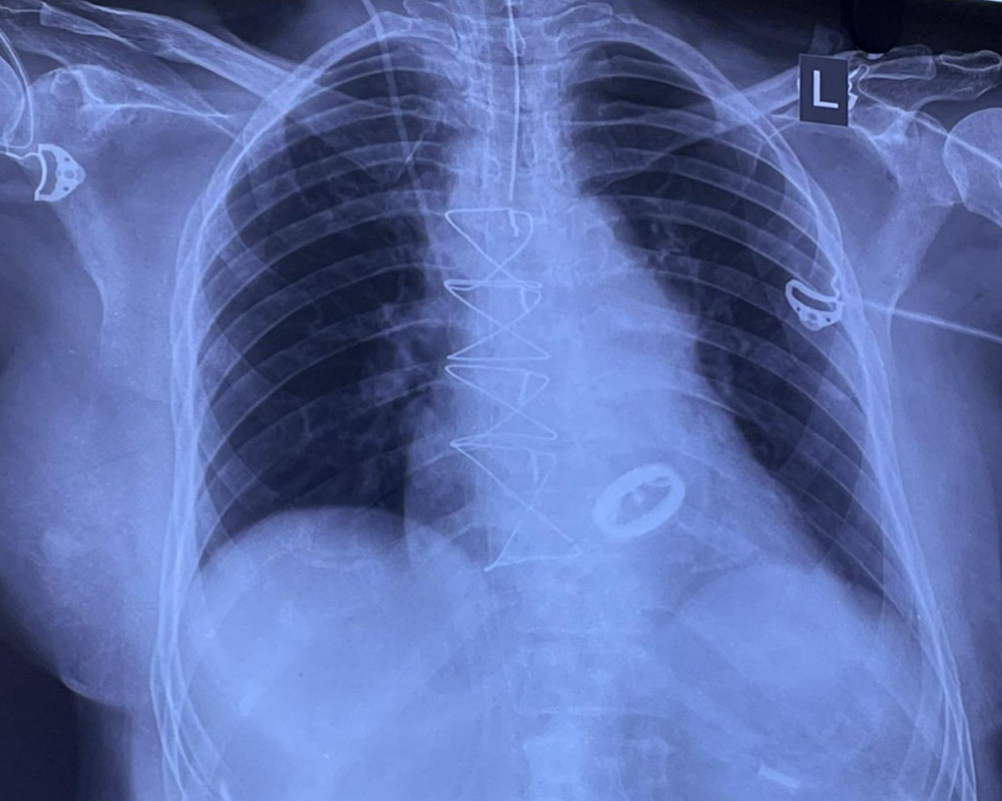
Postoperative chest X-ray showing a mitral prosthesis in situ with intact sternal rings.

Chest radiography was performed after re-exploration that showed intact sternal rings, with well seated mitral prosthesis ( Figure 9). The patient was stable and transferred to the intensive care unit. Repeat blood investigations were performed, and the output was monitored. The serum creatine and electrolyte levels were within the reference limits. She was advised to undergo physiotherapy rehabilitation as supportive care and was discharged a week later.

### Outcome and follow-up

The patient was stable on discharge and was asked to monitor the INR and revisit the physician after 2 weeks post-discharge. On noticing signs of bleeding or worsening symptoms, revisit to the hospital was advised. Patient was evaluated for wound healing, functional recovery and site complications. Physical therapy was advised for mobility. Psychological support was advised if observed for behavioural changes or issues with coping with recovery. Postoperative echocardiography showed a well-functioning prosthesis and resolution of the clot on the RA side. The patient was symptomatically better (NYHA class I) during the first follow-up at 15 days post-discharge. Chest radiography showed prosthesis in situ with no signs of pericardial or pleural effusion. Currently, she is under regular follow-up and is being screened for prosthetic valve function and symptoms.

## Discussion

Postoperative valve-related complications commonly include site and mediastinal bleeding, prosthetic valve dysfunction, prosthetic valve endocarditis, and thromboembolic events. ^3^ Complications are elements of valvular surgery that require intense follow-up; hence, echocardiography plays a vital role in the detection of early complications. However, British guidelines recommend echocardiographic imaging soon after implantation to detect early valve deterioration and plan accordingly on the timing for redo intervention. ^4^ From the point of care, repeat echocardiography is indicated at a frequency that depends on the underlying pathology in order to assess LV performance and pericardial effusion postoperatively.

Echocardiography is indicated if there is a suspicion based on the new onset of symptoms that commonly include breathlessness, fever, or clinical findings with a new onset of murmur. ^5^ Patients undergoing valve replacement require careful postoperative follow-up. Unlike the abovementioned complications, this case had an unusual presentation of extracardiac/intrapericardial hematoma that extrinsically compressed the right atrium with no evidence of pericardial tamponade, an immediate postoperative complication within 24 hours of valve replacement. Huang HD et al. reported a similar case of intrapericardial hematoma presenting with isolated right atrial tamponade, wherein the diagnosis was confirmed by computed tomography (CT) followed by transthoracic echocardiography. ^6^

Bleeding post-cardiac surgery is a complication, often associated with complex procedures causing hemodynamic instability necessitating reexploration for bleeding or tamponade. ^1^ The current patient experienced bleeding at the suture line, thus triggering clot formation attributable to surgical act per se. Bleeding otherwise is commonly witnessed in the majority of the patients undergoing cardiac surgery as aggressive anticoagulation is used during the procedure. Postoperative complications like hematomas are often overlooked if they are located outside of the imaging plane. These complications are often missed and can be misinterpreted for anatomical variants. Therefore, close monitoring for hemodynamic fluctuations and non-invasive imaging are necessary to minimize unnecessary cardiac reexploration requiring cross-clamping and avoid prolonged CPB.

Clinical presentation in patients with hematoma may range from asymptomatic presentation to symptomatic with hemodynamic instability. A case study by Nil Özyüncü ^7^ reported an unusual case of hematoma (LA and LV side) inducing constrictive pericarditis in a patient who underwent aortic valve replacement and bypass grafting, a late complication with the diagnosis confirmed by 2D echocardiography and CT thorax. Hematoma although was evident during the post-operative period, did not induce hemodynamic instability during follow-up visits, expected to be resorbed over the course, therefore not considered for reexploration. Patients with haemorrhagic pericardium in their post-operative period are at a high risk of future constrictive pericarditis for those treated with warfarin and preserved LVEF.

In contrast to the previous case, the current study observed an acute complication, showing a similar presentation with a hematoma around the right atrium due to suture line bleeding. This hematoma was difficult to differentiate from surrounding tissues on TTE and TEE, making it challenging to determine whether it was intrapericardial or extracardiac. Although, the hemodynamics were stable the need for echocardiography per se was indicated for a postoperative evaluation and not based on any clinical suspicion made. This was an incidental diagnosis with an immediate complication as a result of cardiac surgery.

The site of operation is often considered to be the precursor for hematomas. Moreover, etiologies for such hematomas include atrial wall rupture, dissection of aneurysmal aorta, or atrial infarction. Fewer complications of atrial hematomas of unknown causes have also been documented in the literature. ^8^

Although 2D TEE imaging is considered standard imaging during cardiac procedures, it carries greater sensitivity to detect mass and low specificity in differentiating it from intracardiac versus extracardiac. 2D TEE has drawbacks in differentiating anatomical structures at proximity compared to 3D TEE. However, TEE did not play a vital role in delineating the location accurately. The assumptions favoured mass more of extracardiac in location than intracardiac. In a similar case reported by Vladimir V. Merenkov et al. ^9^ highlighted the role of bedside 2D transthoracic imaging in a hemodynamically unstable patient with a retrosternal hematoma mimicking features of tamponade after aortic valve replacement. However, the precursor for the formation of hematoma is not elucidated here. The central hemodynamic instability necessitated the need for transthoracic echocardiography in this context.

Cases on atrial hematomas have been reported in the literature with worse hemodynamic syndrome and clinical presentation. A study by José R. Ortega ^10^ reported 5 clinical cases of atria hematomas in post-valve replacement patients (4 with RA involvement, and 1 LA involvement) with hemodynamic compromise necessitating TEE in locating and guiding reintervention. All 5 cases reported presented with hemodynamic deterioration within 48 hours of the postoperative period, attributed to surgical procedure or associated with coagulation disorders.

The preoperative echocardiography showed the absence of intracardiac mass providing indirect evidence of an acute event causing hematoma formation. Upon reexploration, the mass was located substantially over the right atria requiring evacuation. Thus, the need for reexploring the heart and the need for CPB was avoided. The above statement signifies possible complications faced in immediate post-op intervention wherein it is crucial to identify the lesion by non-invasive imaging being aware of the possible limitations it carries leading to misinterpretation of findings. To conclude, complications witnessed in this case though was an incidental finding, requires a thorough examination, requiring reintervention or conservative management by estimating the benefits over risk. The need for reopening the heart was avoided by promptly identifying and correcting the misinterpretation the of the mass as extracardiac.

Therefore, a thorough echocardiographic examination from multiple windows postoperatively is essential, as mediastinal bleeding involves multifactorial mechanisms. This case report highlights the importance of promptly identifying hematomas that are often missed postoperatively. Early diagnosis can prevent the need for reexploring the heart and prolonged cardiopulmonary bypass time, which are associated with high mortality and morbidity. However, TEE during the perioperative period did not significantly aid in the diagnosis due to its imaging limitations. In the current scenario, 3D TEE could have been highly beneficial in distinguishing the location of the hematoma due to its superior spatial resolution. Also, a multicentric study on similar case reports, can improve the sample size and improve the robustness of the study. To conclude, the indication and type of imaging rely on the clinical suspicion of a complication, its suitability and limitations.

### Patient perspective

“Her initial diagnosis of the disease was made at a tertiary care hospital. We explained the disease severity and prognosis, if not treated. Her symptoms worsened, limiting her daily activities. Initially, we were concerned about the major surgery, but post counseling, we decided to move ahead. We were explained about the procedural complications by the surgeon. Postsurgical nursing care was helpful in her early recovery. The initial days after discharge were difficult, but with constant family support, she was able to adjust to the environment and perform routine activities independently. We are very thankful to the hospitality and care provided by the health care staffs throughout her stay”.

Details provided by patient’s son.

### Ethics and consent

Ethical approval was not required as it is case report. Written informed consent was acquired from the patient’s representative, with approval for waiver granted by the ethics committee. The patient’s guardian has granted consent for publication of the case details while ensuring participant anonymity. The patient has also agreed to the submission of the case report to the journal.

## Data Availability

No data were associated with this article. Figshare: “Case Report: Post-Operative Mitral Valve Replacement Complicating with a Large Cardiac Mass and Role of TEE in Decision Making”, figshare data, 10.6084/m9.figshare.25547809.v1. ^11^ This project contains the following underlying data:
• Date file 1: Case images • Date file 2: Media files and Care checklist Data are available under the terms of the Creative Commons Attribution 4.0 International license (CC-BY 4.0).
