# Supplementary material for: Case Report: Post-operative mitral valve replacement complicating with a large cardiac mass and the role of TEE in Imaging
Source: F1000Res. 2024 Oct 1;13:306. Originally published 2024 Apr 22. [Version 2] doi: 10.12688/f1000research.145007.2 (PMC11489830; doi:10.12688/f1000research.145007.2)
Supplement: Peer review history

# Reviewer report: Reviewer response for version 2

**DOI:** 10.5256/f1000research.171807.r328387

**Published:** 2024-10-18

**Authors:** Raif Kılıç

**Affiliations:** 1 Çermik State Hospital, Diyarbakır, Turkey

I have reviewed the article titled ''Case Report: Post-operative mitral valve replacement complicating with a large cardiac mass and the role of TEE in Imaging''. As a result of my evaluation, I found the scientific content and methods of the manuscript appropriate and recommend its indexing in bibliographic databases. I believe that the study will contribute to the literature.

 Thank you and I am grateful for giving me the opportunity to review this process.

 Best regards,

 Dr. Raif KILIÇ

Are enough details provided of any physical examination and diagnostic tests, treatment given and outcomes?

Yes

Is the case presented with sufficient detail to be useful for other practitioners?

Partly

Is sufficient discussion included of the importance of the findings and their relevance to future understanding of disease processes, diagnosis or treatment?

Yes

Is the background of the case’s history and progression described in sufficient detail?

Yes

Reviewer Expertise:

Heart valve diseases, heart failure, arrhythmia, interventional cardiology

I confirm that I have read this submission and believe that I have an appropriate level of expertise to confirm that it is of an acceptable scientific standard.

# Reviewer report: Reviewer response for version 2

**DOI:** 10.5256/f1000research.171807.r328386

**Published:** 2024-10-09

**Authors:** Adem Aktan

**Affiliations:** 1 Mardin Artuklu University Faculty of Medicine, Mardin, Turkey

I commend the author for the revisions and changes made to the manuscript. The improvements are constructive and enhance the overall quality of the work. I recommend accepting the manuscript in its current form

Are enough details provided of any physical examination and diagnostic tests, treatment given and outcomes?

Partly

Is the case presented with sufficient detail to be useful for other practitioners?

Partly

Is sufficient discussion included of the importance of the findings and their relevance to future understanding of disease processes, diagnosis or treatment?

Partly

Is the background of the case’s history and progression described in sufficient detail?

Partly

Reviewer Expertise:

I commend the authors for the revisions and changes made to the manuscript. The improvements are constructive and enhance the overall quality of the work. I recommend accepting the manuscript in its current form.

I confirm that I have read this submission and believe that I have an appropriate level of expertise to confirm that it is of an acceptable scientific standard.

# Reviewer report: Reviewer response for version 1

**DOI:** 10.5256/f1000research.158882.r291828

**Published:** 2024-07-01

**Authors:** Raif Kılıç

**Affiliations:** 1 Çermik State Hospital, Diyarbakır, Turkey

In this case report, the authors presented a case example describing a postoperative cardiac mass and the role of TEE in the diagnosis of this mass. I congratulate the operators as it was a very well managed complication. However, I will have some revision suggestions in the article;

 -To what do you attribute the formation of the post-op mass? Inadequate anticoagulation?, coagulopathy?... It is recommended to add this to the article.

 - In the TEE performed after the procedure, could it be determined whether the mass was outside the atrium or not? If not, then how can it be superior to TTE? Could imaging with TTE, which is easier to perform, instead of TEE, be sufficient?

 - Cerebrovascular events were mentioned in the discussion section, but they are not related to the topic. Removal or correction is recommended.

Are enough details provided of any physical examination and diagnostic tests, treatment given and outcomes?

Yes

Is the case presented with sufficient detail to be useful for other practitioners?

Partly

Is sufficient discussion included of the importance of the findings and their relevance to future understanding of disease processes, diagnosis or treatment?

Yes

Is the background of the case’s history and progression described in sufficient detail?

Yes

Reviewer Expertise:

Heart valve diseases, heart failure, arrhythmia, interventional cardiology

I confirm that I have read this submission and believe that I have an appropriate level of expertise to confirm that it is of an acceptable scientific standard, however I have significant reservations, as outlined above.

# Response

**Published:** 2024-09-17

**Authors:** chaithra Nayak

**Affiliations:** Cardiology, Manipal Academy of Higher Education, Manipal, Karnataka, India

Dear reviewer,

 We thank you for your valuable time and comments

 Please find the responses to the comments received 

 Dear Dr. Raif Kılıç

 Thank you for your thoughtful and insightful review of our paper. 

 The following are the responses to the comments received

 1. **To what do you attribute the formation of the post-op mass? Inadequate anticoagulation? coagulopathy?... It is recommended to add this to the article.**

 Response Comment: Thank you for your valuable comments

 In the current case, we attribute hematoma formation to suture line bleeding. As per the recommended protocol, the patient was adequately anticoagulated during the perioperative period. We believe that such complications witnessed irrespective of the underlying etiology must be addressed for necessary management.

 2. **In the TEE performed after the procedure, could it be determined whether the mass was outside the atrium or not? If not, then how can it be superior to TTE? Could imaging with TTE, which is easier to perform, instead of TEE, be sufficient?**

**Response comment:** We appreciate your time and valuable comments in this context

 After carefully reviewing the comments, we would like to highlight the challenge we encountered in assessing the utility of TEE in imaging. One significant limitation of both TTE and TEE is their ability to characterize tissue and accurately determine the location of a mass. In this particular case, TEE did not play a significant role in distinguishing whether the mass was intra- or extracardiac, highlighting a limitation of the imaging modality (Page no.9 paragraph 4, Page no.10, para 1).

**3. Cerebrovascular events were mentioned in the discussion section but are unrelated to the topic. Removal or correction is recommended.**

 Response comment: We are grateful for your time and deep insights into this paper. The necessary modifications have been carried out and upgraded with the recent references.

# Reviewer report: Reviewer response for version 1

**DOI:** 10.5256/f1000research.158882.r291827

**Published:** 2024-06-19

**Authors:** Adem Aktan

**Affiliations:** 1 Mardin Artuklu University Faculty of Medicine, Mardin, Turkey

This case report meticulously address a rare complication following cardiac valve replacement, offering significant clinical insights. However, several refinements can be suggested for the manuscript:

**1.** The introduction should commence with an emphasis on the definition and implications of an intrapericardial hematoma, rather than starting with a general overview of postoperative complications.

**2.** In the case report, clinical findings and diagnostic imaging results should be presented in a more coherent sequence. There should be a clear linkage established between clinical observations and radiological evaluations.

**3.** The section on Transoesophageal Echocardiography (TEE) could more prominently underscore how TEE guided decision-making and its critical role in patient management.

**4.** Additional details are warranted regarding postoperative management strategies and follow-up protocols.

**5.** In the discussion section, recommendations can be provided on how similar case reports can be integrated into the literature and suggestions can be made for managing such rare complications.

**6.** The conclusion should succinctly highlight the clinical significance of the case report and its implications for future clinical practice and management of similar cases.

**7.** References 1, 9, and 10 should be updated with more recent sources.

Are enough details provided of any physical examination and diagnostic tests, treatment given and outcomes?

Partly

Is the case presented with sufficient detail to be useful for other practitioners?

Partly

Is sufficient discussion included of the importance of the findings and their relevance to future understanding of disease processes, diagnosis or treatment?

Partly

Is the background of the case’s history and progression described in sufficient detail?

Partly

Reviewer Expertise:

This case report comprehensively address a rare complication and provide significant clinical insights. However, publication following the suggested improvements in the manuscript is appropriate.

I confirm that I have read this submission and believe that I have an appropriate level of expertise to confirm that it is of an acceptable scientific standard, however I have significant reservations, as outlined above.

# Response

**Published:** 2024-09-17

**Authors:** chaithra Nayak

**Affiliations:** Cardiology, Manipal Academy of Higher Education, Manipal, Karnataka, India

Mrs. Chaithra Nayak, Department of Cardiology, KMC, Mangalore 

 Dear reviewers,

 We thank you for your valuable time and comments

 Please find the responses for the comments received attached with a response sheet

 Dear Dr. Adem Aktan

 Thank you for your thoughtful and insightful review of our paper. 

 1. **The introduction should commence with an emphasis on the definition and implications of an intrapericardial hematoma, rather than starting with a general overview of postoperative complications.**

**Response comments: **This was truly a valuable comment. We have incorporated the necessary information related to the definition of the complication witnessed in the case report and have carried out the essential revisions as stated above (Page no. 4, paragraph 1).

 2. **In the case report, clinical findings and diagnostic imaging results should be presented in a more coherent sequence. There should be a clear linkage established between clinical observations and radiological evaluations**

**Response comments**: ****We sincerely appreciate your comments and observations. We have made the necessary revisions to better communicate the clinical findings (Page no. 5, paragraph 1,2).

 3. **The section on Transesophageal Echocardiography (TEE) could more prominently underscore how TEE guided decision-making and its critical role in patient management.**

**Response comments: **Thank you for your valuable comments and suggestions. After thoroughly reviewing the manuscript and checking for the reviewer's comments, we strongly feel a disparity in projecting TEE as a superior imaging tool. TEE, however, played a vital role in imaging the complication in this current study. However, due to its limitations, did not yield greater benefits in determining the location. Therefore, its utility in decision-making is less highlighted here (Page no.9, paragraph 4).

**4. Additional details regarding postoperative management strategies and follow-up protocols are warranted.**

**Response comments: **Thank you for the suggestions. The above-stated points are critically reviewed by us. The management strategies are aligned and post-operative follow-up data are adequately covered under the section Outcome and follow-up (Page no.10, paragraph 1,2,3).

**5. In the discussion section, recommendations can be provided on how similar case reports can be integrated into the literature and suggestions can be made for managing such rare complications**

**Response comments:** We appreciate your time in thoroughly revising this paper. Findings related to the management of postoperative complications for similar case studies are included and their novel treatment approaches for such similarly reported cases (Page no.8, paragraph 2; Page no.9, paragraph 1,2).

 6. **The conclusion should succinctly highlight the clinical significance of the case report and its implications for future clinical practice and management of similar cases.**

**Response comments: **We value your feedback points upon reviewing the paper. We have thoughtfully added the points highlighting the clinical importance and the need for imaging which is crucial to monitor such complications. The above-mentioned comments with necessary corrections have been implemented in this paper (Page no. 11,12,13).

**7.** **References 1, 9, and 10 should be updated with more recent sources.**

**Response comment**: We are thankful for the in-depth revision. The necessary corrections have been carried out (Page no 15,16).
